# Processing Strategy and Comparative Performance of Different Mobile LiDAR System Grades for Bridge Monitoring: A Case Study

**DOI:** 10.3390/s21227550

**Published:** 2021-11-13

**Authors:** Yi-Chun Lin, Jidong Liu, Yi-Ting Cheng, Seyyed Meghdad Hasheminasab, Timothy Wells, Darcy Bullock, Ayman Habib

**Affiliations:** 1 Lyles School of Civil Engineering, Purdue University, West Lafayette, IN 47907, USA; lin934@purdue.edu (Y.-C.L.); liu2845@purdue.edu (J.L.); cheng331@purdue.edu (Y.-T.C.); hashemin@purdue.edu (S.M.H.); darcy@purdue.edu (D.B.); 2 Indiana Department of Transportation Research and Development, West Lafayette, IN 47907, USA; twells@indot.in.gov

**Keywords:** bridge evaluation, infrastructure inspection, as-built data, bridge deck thickness, mobile LiDAR, registration, planar/linear/cylindrical features

## Abstract

Collecting precise as-built data is essential for tracking construction progress. Three-dimensional models generated from such data capture the as-is conditions of the structures, providing valuable information for monitoring existing infrastructure over time. As-built data can be acquired using a wide range of remote sensing technologies, among which mobile LiDAR is gaining increasing attention due to its ability to collect high-resolution data over a relatively large area in a short time. The quality of mobile LiDAR data depends not only on the grade of onboard LiDAR scanners but also on the accuracy of direct georeferencing information and system calibration. Consequently, millimeter-level accuracy is difficult to achieve. In this study, the performance of mapping-grade and surveying-grade mobile LiDAR systems for bridge monitoring is evaluated against static laser scanners. Field surveys were conducted over a concrete bridge where grinding was required to achieve desired smoothness. A semi-automated, feature-based fine registration strategy is proposed to compensate for the impact of georeferencing and system calibration errors on mobile LiDAR data. Bridge deck thickness is evaluated using surface segments to minimize the impact of inherent noise in the point cloud. The results show that the two grades of mobile LiDAR delivered thickness estimates that are in agreement with those derived from static laser scanning in the 1 cm range. The mobile LiDAR data acquisition took roughly five minutes without having a significant impact on traffic, while the static laser scanning required more than three hours.

## 1. Introduction

Securing bridges with good structural and functional conditions starts with effective quality assurance and quality control during construction to ensure that such infrastructure fully satisfies the design requirements [1]. In addition, frequent and accurate inspection of the structural and functional conditions of each bridge is required to ensure traffic safety and prioritize maintenance [2]. Traditional bridge evaluation practices rely on visual inspection and point-based measurements. Deck thickness is typically checked during and post-construction to ensure structural adequacy and conformance. For instance, the American Association of State Highway and Transportation Officials (AASHTO) requires that the minimum thickness of a concrete deck should not be less than 7 inches [3]. To evaluate bridge deck thickness during construction, measurements are typically taken from a string line pulled between the screed rails or a pole stabbed into the plastic concrete—i.e., freshly poured concrete [4]. Post-construction, ultrasonic thickness meters are usually used. Such approaches are labor-intensive and prone to human errors, as they require trained personnel to identify structurally unsound locations. Furthermore, this spot sampling is not sufficient to capture the thickness values over the entire bridge, which are necessary to provide as-built documentation that can be used for long-term asset inventory and management.

Modern remote sensing techniques provide promising non-contact alternatives for during- and post-construction bridge evaluation and inspection. Prior research has established principles and procedures for using unmanned aerial vehicle (UAV) imagery for bridge visual inspection and 3D information reconstruction [5,6,7,8,9]. Moreover, with several protection mechanisms, UAVs can maneuver at a close proximity to the bridge and even perform contact inspection tasks [10,11,12]. In contrast to imaging sensors, light detection and ranging (LiDAR) provides direct 3D measurements that can be used for quantitative evaluation. Terrestrial laser scanners (TLSs) can acquire high-resolution point clouds with millimeter-level precision, deemed as the gold standard in construction management and infrastructure monitoring. However, the time and labor required for data acquisition and post-processing increase significantly for a large site. The scanner might have to be set up within the driving lane to acquire sufficient point density along the structure, which in turn would affect traffic flow. Therefore, monitoring large-sized infrastructure using TLSs might not be practical and/or scalable. Mobile LiDAR mapping systems (MLMSs) facilitate efficient field surveys that can cover large areas with a minimal impact on traffic flow. For MLMSs, direct georeferencing, i.e., trajectory information provided by the onboard global navigation satellite system/inertial navigation system (GNSS/INS) unit, is typically adopted to reconstruct point clouds in a common reference/mapping frame. Point cloud quality therefore depends on the ranging accuracy of LiDAR units, the grade of the onboard GNSS/INS unit, and the reliability of the system calibration procedure. Centimeter-level positional accuracy can be expected if the quality of derived georeferencing data from the GNSS/INS unit and system calibration parameters are guaranteed. However, intermittent access to a GNSS signal and issues pertaining to system calibration cloud results in discrepancy of more than several centimeters between point clouds from different drive runs/flight lines. In this case, registration is required to fine-tune the point cloud alignment.

This paper describes an assessment of alternative mobile LiDAR systems for bridge monitoring. More specifically, this study addresses the following research questions: (i) can we use MLMS data (with centimeter-level positional accuracy) to derive quantitative measures of bridge and achieve an accuracy similar to that from TLS data, and (ii) are surveying-grade MLMS systems (with millimeter- to centimeter-level accuracy) better than mapping-grade MLMS systems (with an accuracy of a few centimeters) in terms of providing quantitative assessments of bridges? The key contributions of this study can be summarized as follows:

- A semi-automated feature-based fine registration is proposed to compensate for the impact of georeferencing and system calibration errors on mobile LiDAR data. The developed registration strategy can also be used to fine-tune the point cloud alignment among different TLS scans.
- A bridge deck thickness evaluation strategy based on surface-to-surface distance is proposed to minimize the impact of inherent noise on the point clouds. The aim is to achieve an accuracy better than ±1 cm for the derived thickness measures.
- The performance of different MLMS grades is assessed against TLS data in terms of the quality of derived thickness measures, scalability, and impact on traffic flow.

The remainder of this paper is structured as follows: Section 2 provides an overview of prior research; the mobile mapping systems, study site, and data collection procedure are introduced in Section 3; the feature-based registration and bridge deck thickness evaluation approaches are covered in Section 4; the results together with their analysis are discussed in Section 5, which is followed by a summary of the study conclusions and recommendations for future research in Section 6.

## 2. Related Work

### 2.1. LiDAR for Infrastructure Mapping

LiDAR, known for its ability to directly generate accurate 3D point clouds with high density, has recently been receiving an increasing amount of interest by the construction management and infrastructure monitoring research/professional communities. Acquired point clouds by TLSs have been used for generating precise 3D models to evaluate the progression of construction processes [13,14,15]. The high accuracy of TLS point clouds also allows for millimeter-level displacement and deformation evaluation post-construction [16,17,18]. However, the point density and accuracy of TLS point clouds drop quickly as the distance from the sensor increases. To ensure full coverage of the structure in question, multiple TLS stations are required. The station locations should be carefully chosen to minimize occlusions and have sufficient overlap (i.e., common areas) for the registration of point clouds from different scans. For a large site, time, labor, and location requirements for data acquisition increase significantly, making it unrealistic to apply such a technique in a scalable manner.

Mobile LiDAR has emerged as a promising alternative that can overcome the shortcomings of TLSs. One or more LiDAR units can be mounted on various platforms, e.g., UAVs, trucks, tractors, and robots. Field surveys with mobile LiDAR are efficient and can cover large areas that are impractical to conduct with TLSs. These key benefits have stimulated the interest of the research/professional community to apply mobile LiDAR for analyzing complex road environments, such as lane marking detection and road boundary extraction [19,20,21,22], as well as mapping railroads and tunnels [23,24,25,26]. Several studies have validated and reported the accuracy of mobile LiDAR data for monitoring civil infrastructure. Puri and Turkan [27] used a wheel-based mobile LiDAR system equipped with a Velodyne HDL-64E LiDAR unit for tracking the construction progress of a bridge. They pointed out that a noise level in the range of ±3–4 cm was present in the as-built data from MLMSs, affecting the performance of progress tracking. Lin et al. [28] evaluated the performance of a wheel-based MLMSs equipped with four Velodyne LiDAR units for mapping airfield pavement before and after a resurfacing process. In their study, the positional accuracy of LiDAR point clouds was in the ±5 cm range, and a 9 cm increase in pavement elevation after resurfacing was detected. Another study verified the absolute accuracy of point clouds acquired by a commercially available system, the Lynx Mobile Mapper system from Optech Inc. (Toronto, ON, Canada) [29]. Their test site was a university campus, comprised of both infrastructure and vegetation. Although the range accuracy of the onboard LiDAR unit in that study was ±8 mm, the absolute accuracy of the point cloud was determined to be in the range of ±1 to ±5 cm, mainly attributed to the trajectory quality. Moreover, in areas with limited/intermittent access to GNSS signals, the positional accuracy might deteriorate to the ±0.3 m range. Inaccuracy of the system calibration parameters would cause additional deterioration in the derived point clouds. Overall, millimeter-level positional accuracy is difficult to achieve even with high-end LiDAR units due to GNSS reception issues and/or system calibration artifacts. Data processing and analysis strategies that reduce the impact of the above factors, as well as inherent noise in the point cloud, are required to take full advantage of potential benefits of mobile LiDAR.

### 2.2. Point Cloud Registration

Point cloud registration aims at aligning LiDAR data from different TLS scans and/or MLMS drive runs/flight lines to a common reference frame. Registration approaches can be categorized based on the used primitives; namely, direct cloud-to-cloud registration and feature-based registration. The well-known iterative closest point (ICP) [30,31] and its variants belong to the cloud-to-cloud registration category. Such algorithms assume that some initial transformation parameters exist, and their aim is to refine these parameters. Besl and McKay [30] assumed that the closest points from two point clouds after coarse registration constitute a conjugate point pair, and used such pairs for the estimation of transformation parameters. Chen and Medoini [31] further considered surfaces normal during point matching; more specifically, point-to-point correspondence is established by normally projecting a point in one scan onto its adjacent surface in the other scan. Several variants were introduced to improve the ICP robustness using local neighborhood characteristics [32,33]. Studies that share similar concepts, such as the iterative closest projected point [34], point-to-plane registration [35], and multiscale model-to-model cloud comparison [36] have been introduced. In addition, some registration algorithms start by generating a 3D mesh or surface model (e.g., triangulated irregular network), and then use cloud-to-surface or surface-to-surface pairings to estimate the transformation parameters [37,38]. However, creating a mesh is a complex task, especially for point clouds with vertical discontinuities and/or excessive occlusions. In general, cloud-to-cloud fine registration using the ICP or its variants has some disadvantages: (i) it requires large overlap areas between point clouds; (ii) it is sensitive to point density distribution and noise level within the point clouds; and (iii) it requires solid surfaces with good variation in orientation/slope/aspect. For TLS and mobile LiDAR data, a large overlap is not always guaranteed due to the varying sensor-to-object distance, occlusions, and constraints imposed by the scanning environment (e.g., traffic along transportation corridors). In addition, the point density and precision drop quickly as one moves away from the sensor/trajectory, and the distribution of the surface orientation variation within the study site can be unbalanced. These factors could lead to overweighting when estimating the transformation parameters. In order to meet high accuracy requirements, user intervention is unavoidable. 

Another group of registration algorithms utilize common features that can be identified in point clouds captured at different locations. In general, such algorithms do not require coarse alignment of the point clouds. The major task in feature-based registration is the identification of common points/features. This task can be quickly performed semi-automatically. Special targets (e.g., highly reflective checkerboard and/or spherical targets), which can be identified in the point clouds, were commonly used to increase the level of automation within the registration process [39,40,41,42]. Among such targets, spherical targets were used more frequently since they eliminate the need to re-orient the target to face the scanner during point cloud acquisition [40]. Target-free registration is nonetheless the ultimate goal and, therefore, many studies focused on the automated identification and matching of features. In urban areas, geometric primitives such as point, linear, cylindrical, and planar features can be reliably extracted from point clouds. Such primitives have been employed to estimate the transformation parameters between two point clouds [41,43,44,45,46,47,48,49,50,51,52]. Some studies demonstrated the ability of feature-based registration in handling point clouds acquired by different platforms, including airborne LiDAR, mobile LiDAR, TLSs, and even imagery [53,54,55,56,57,58]. Habib et al. [59,60] registered LiDAR and photogrammetric data using linear features. Renaudin et al. [61] utilized photogrammetric data to help in feature extraction and the registration of TLS data with minimal overlap. In addition to geometric primitives, key points based on local feature descriptors that encompass local shape geometry were used for registration [62,63,64]. Recently, learning-based local feature descriptors and point cloud registration framework targeting fully automated feature detection, and matching evolved rapidly [65,66,67,68,69,70,71]. While deep-learning-based methods have shown superior performance on several benchmark datasets, their generalization ability on unseen real datasets needs careful evaluation.

Currently, the vast majority of existing registration tasks are based on pair-wise registration (i.e., the registration process is sequentially established two scans at a time) and/or require initial segmentation to derive the feature parameters, which are used in the registration process. Pair-wise registration has two disadvantages: (i) it makes the process time-consuming when dealing with multiple scans and/or drive-runs; and (ii) the sequential registration leads to the propagation of errors, which increases as we move away from the reference scan. Furthermore, existing algorithms commonly utilize feature parameters (e.g., line endpoints, direction vector of linear/axis of cylindrical features, and normal vector of a plane) for the registration process [40,42,43,44,45,46,47,48,49,50,51,52,53,54,55,56,57,58,59,60]. Direct use of these parameters would not allow for the sufficient mitigation of inherent noise and/or point density variations in the point cloud. In response to these challenges, this study provides a strategy for the simultaneous registration of several scans from TLS and MLMS drive runs. Moreover, the individual points along the registration features, rather than their parameters, are used in the registration process, thus allowing for the mitigation of noise level/point density variation in the point cloud as well as the negative impact of the partial coverage of registration primitives in the different scans.

## 3. Data Acquisition Systems and Field Surveys

In this study, MLMS and TLS datasets were collected over and underneath a highway bridge that was suspected to have an inadequate deck thickness. This section introduces the data acquisition system and calibration strategy. We also describe the study site and provide information regarding field surveys.

### 3.1. System Description and Calibration

**Figure 1:**
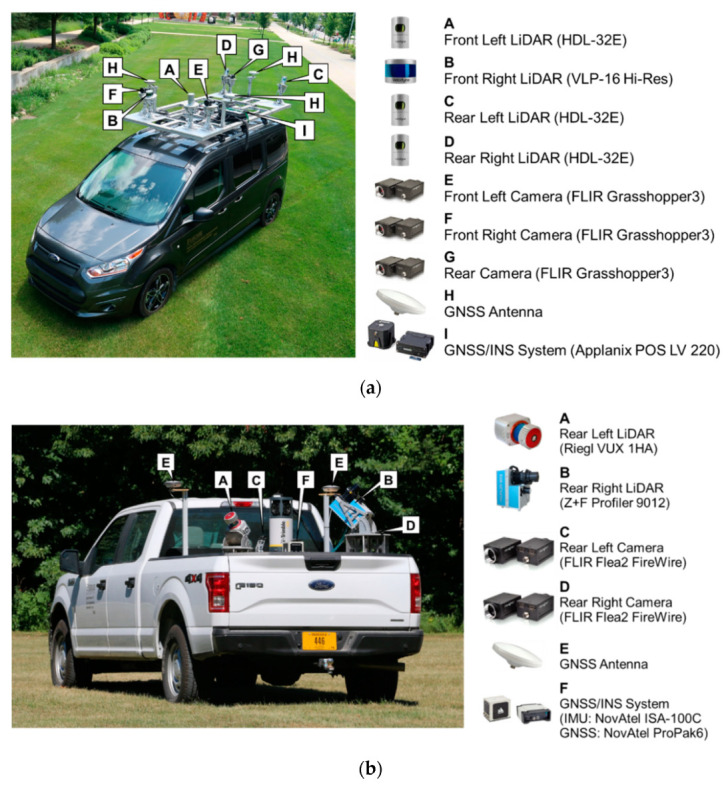
The wheel-based mobile mapping systems and onboard sensors used in this study: (**a**) Purdue Wheel-based Mobile Mapping System-High Accuracy (PWMMS-HA) and (**b**) Purdue Wheel-based Mobile Mapping System-Ultra High Accuracy (PWMMS-UHA). Both platforms are non-commercial systems designed and integrated by the research group.

The MLMS data used in this research were captured by a mapping-grade system—Purdue Wheel-based Mobile Mapping System-High Accuracy (PWMMS-HA)—and a surveying-grade system—Purdue Wheel-based Mobile Mapping System-Ultra High Accuracy (PWMMS-UHA). Figure 1a shows the PWMMS-HA, which is equipped with four LiDAR units (three Velodyne HDL-32E and one Velodyne VLP-16 High Resolution), three cameras, and a GNSS/INS unit for direct georeferencing. The front-left, front-right, rear-left, and rear-right LiDAR units are hereafter denoted as HDL-FL, VLP-FR, HDL-RL, and HDL-RR, respectively. The range accuracy of the Velodyne HDL-32E and VLP-16 is ±2 cm and ±3 cm, respectively [72,73]. The post-processing positional accuracy of the GNSS/INS unit is ±2 cm with an attitude accuracy of 0.020° and 0.025° for the roll/pitch and heading, respectively [74]. The PWMMS-UHA, shown in Figure 1b, is outfitted with two 2D profiler LiDAR units: a Riegl VUX-1HA (hereafter denoted as RI) and a Z+F Profiler 9012 (hereafter denoted as ZF). Two rear-looking cameras and georeferencing units are also installed on this system. The range accuracy of the RI and ZF scanners is ±5 mm and ±3 mm, respectively [75,76]. The GNSS/INS post-processing positional accuracy is ±1–2 cm with an attitude accuracy of ±0.003° for pitch/roll and ±0.004° for heading [77].

The rigorous system calibration introduced by Ravi et al. [78] was carried out for both MLMS vehicles to determine the relative position and orientation (denoted hereafter as mounting parameters) between the onboard sensors and IMU body frame, whose position and orientation are derived through the GNSS/INS integration process. The expected post-calibration positional accuracy of the derived point cloud was estimated using the individual sensors’ specifications and standard deviations of the estimated mounting parameters through the LiDAR error propagation calculator developed by Habib et al. [79]. The calculator suggests an expected accuracy of about ±4 cm and ±2 cm at a range of 30 m from the vehicle for the PWMMS-HA and PWMMS-UHA, respectively.

The TLS point clouds were captured by FARO Focus 3D X330 and Trimble TX8b laser scanners. The FARO Focus 3D X330 laser scanner is integrated with a high-dynamic-range imaging unit. It has a range systematic error of ±2 mm and a range noise better than ±0.5 mm for objects 10 m to 25 m away from the scanner (one sigma). The scanning speed is up to 976,000 points per second with a maximum range of 330 m [80]. The Trimble TX8b laser scanner has a range systematic error of ±2 mm and a range noise better than ±2 mm for objects 2 m to 120 m away from the scanner (in standard modes at one sigma). It can scan up to one million points per second with a maximum range of 120 m [81]. 

### 3.2. Study Site and Field Surveys

**Figure 2:**
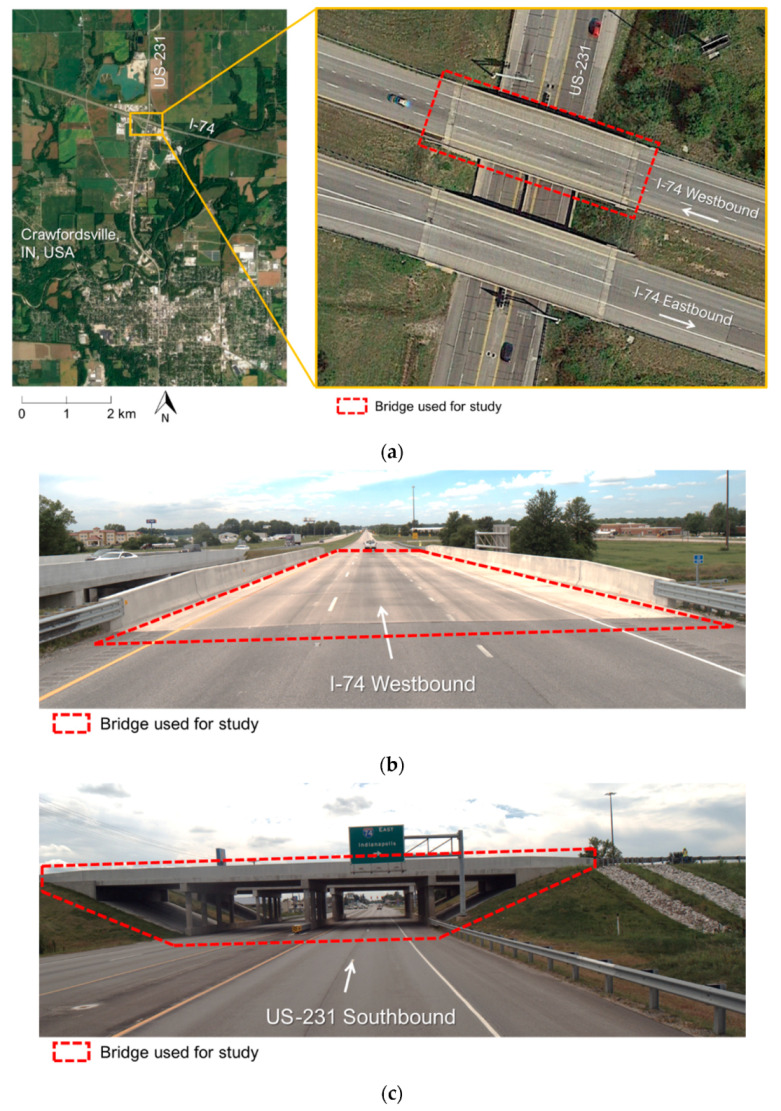
Study site: (**a**) the westbound bridge at the intersection of the I-74 and US-231 near Crawfordsville in Indiana, USA (aerial photo adapted from Google Earth images), (**b**) image of the bridge captured by PWMMS-HA while driving westbound on the I-74, and (**c**) side view of the bridge captured by PWMMS-HA while driving southbound on the US-231.

Field surveys were conducted over and underneath a westbound bridge along an interstate highway at the intersection of the I-74 and US-231 near Crawfordsville in Indiana, USA (shown in Figure 2a). The bridge in question required post construction grinding to meet smoothness and ride quality standards. Once grinding was completed, it was important to perform an as-built survey to document bridge deck thickness. Figure 2b,c displays images acquired by one of the cameras onboard the PWMMS-HA, showing the concrete deck and side view of the bridge, respectively. 

**Figure 3:**
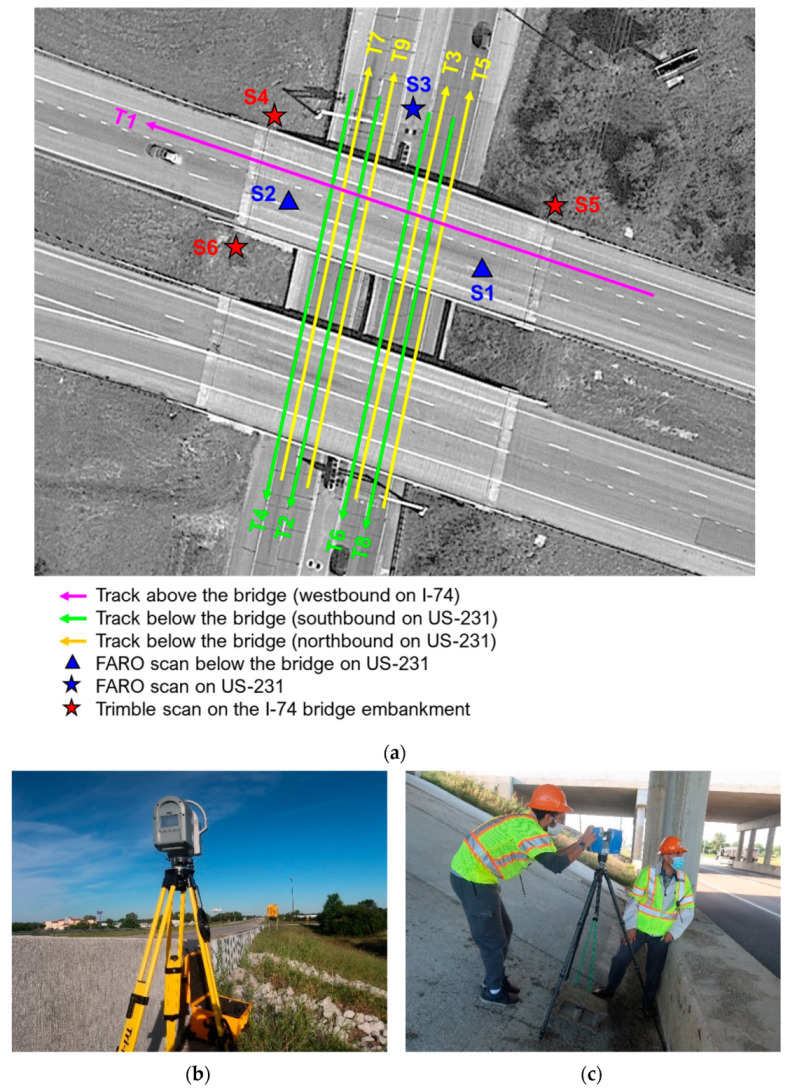
Data acquisition: (**a**) drive run configuration for the vehicles (Tracks T1–T9) and TLS scan locations (Scans S1–S6), (**b**) image of the Trimble station (Scan S4) atop the I-74 embankment outside the barrier rail, and (**c**) image of the FARO station (Scan S1) on the US-231 under the I-74 bridge.

**Figure 4:**
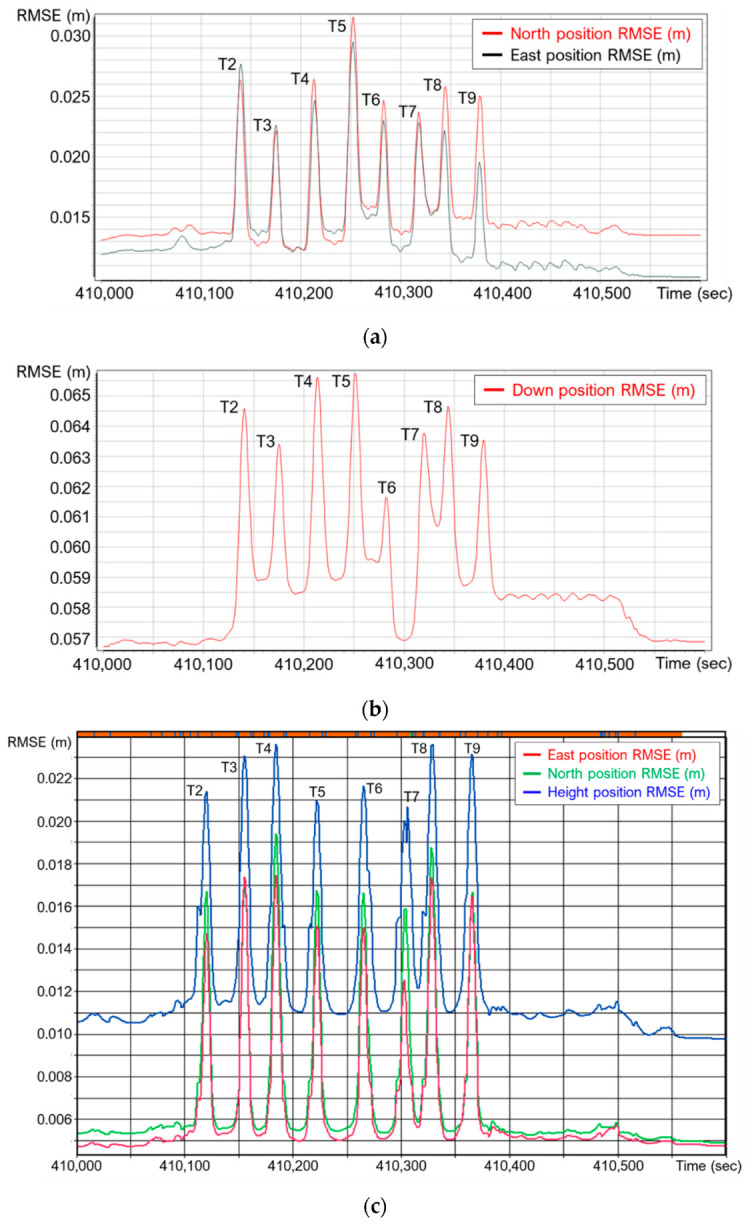
Global navigation satellite system/inertial navigation system (GNSS/INS) position accuracy charts for the (**a**) PWMMS-HA (north and east positions), (**b**) PWMMS-HA (down position), and (**c**) PWMMS-UHA vehicles. The highlighted eight peaks correspond to the eight southbound and northbound tracks (Tracks T2–T9) on the US-231 below the bridge, where suboptimal position accuracy can be observed.

Table 1 lists the specifications of the three datasets collected in this study. The MLMS drive run configuration and TLS scan locations are shown in Figure 3a. Both MLMS vehicles started by driving westbound on the I-74 (magenta track in Figure 3a, T1), and then drove southbound and northbound below the bridge on the US-231 (green—T2, T4, T6, and T8—and yellow—T3, T5, T7, and T9—tracks in Figure 3a). Just before reaching the bridge on the I-74, a highway patrol cruiser slowed down the traffic while driving the PWMMS-UHA and PWMMS-HA. Tracks T2–T9, which were designed for investigating the impact of partial GNSS signal outage, were conducted while stopping the southbound and northbound traffic on US-231 for less than three minutes. In total, the data acquisition on the I-74 and US-231 took about five minutes. The average driving speed was 30 km/h for both MLMS vehicles. Figure 4 illustrates the position accuracy charts reported by the GNSS/INS integration software, providing a glimpse of the trajectory quality for the two MLMS vehicles. As can be observed in the figure, the position error of PWMMS-UHA is smaller compared to that of PWMMS-HA, owing to the higher-end IMU onboard. In Figure 4, the highlighted eight peaks correspond to the eight tracks below the bridge (T2–T9 in Figure 3a), suggesting suboptimal accuracy for the below-bridge tracks due to intermittent access to a GNSS signal. The position error, however, does not increase over time since (i) the signal was restored when the vehicles cleared the bridge, (ii) the GNSS data were processed in both forward and backward directions, and (iii) the relatively short GNSS data outage duration can be handled by the onboard IMU.

To speed up the scanning process, TLS point clouds underneath and over the bridge were simultaneously captured by FARO Focus 3D X330 and Trimble TX8b laser scanners, respectively. Figure 3a illustrates the scan locations with the Trimble stations set up outside the barrier rail atop of the I-74 bridge embankment to capture the top surface of the deck—Figure 3b. The FARO stations were located along the US-231 to capture the bottom surface of the deck—Figure 3c. While the below-bridge FARO scan locations ensured sufficient coverage of the bottom surface of the deck, the setup of the Trimble scans was less than optimal as the overlap region between the east (S5) and west (S4 and S6) scans, which are 55 m apart, happens at the middle of the bridge. Therefore, one might expect less than optimal registration of the Trimble scans due to large separation, lower point density, and shallow scan angles within the overlap region. In terms of data collection time, each TLS scan took about thirty-five minutes for a total of three hours, considering the time for the scanners to move and set up between the different locations.

## 4. Methodology for Point Cloud Registration and Bridge Deck Thickness Evaluation

**Figure 5:**
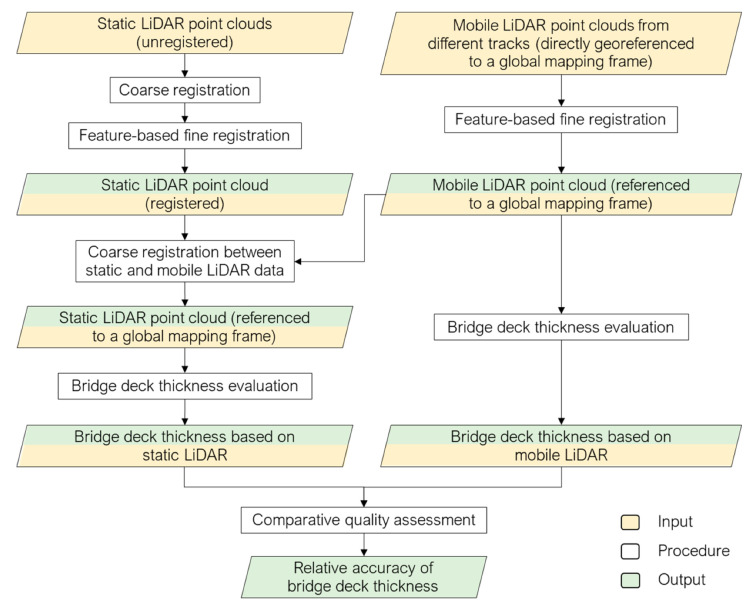
Workflow of the proposed bridge deck thickness evaluation and comparative quality assessment strategy.

The proposed workflow (shown in Figure 5) is comprised of registration, bridge deck thickness evaluation, and comparative quality assessment. For MLMS tracks, the point clouds are directly georeferenced in a global mapping frame, and coarse alignment is thus guaranteed. Feature-based fine registration is carried out to fine-tune the alignment between point clouds from different tracks. For TLSs, the point cloud acquired by each scan is available in a different local reference frame, which is defined by the scan location/setup. A coarse registration is carried out based on manually identified conjugate points in the respective point clouds, so that the feature extraction (which will be covered later) for individual scans can be conducted simultaneously. A successive feature-based fine registration is performed to align the point clouds from the different scans. One should note that whether a point cloud is in a local or global reference frame is not critical for the evaluation of bridge deck thickness. In this study, a coarse registration between the TLS and MLMS data is performed only to compare their thickness estimates. The following subsections describe the proposed feature-based fine registration, bridge deck thickness evaluation, and comparative quality assessment.

### 4.1. Feature-Based Fine Registration

The key advantage of the proposed registration strategy is the simultaneous alignment of point clouds from multiple TLS scans or MLMS drive-runs using planar, linear, and cylindrical features without the setup of specific targets. Besides point cloud alignment, the parametric model of registration primitives is derived and can be used for developing an as-built 3D model as well as the subsequent monitoring of bridge elements (i.e., settlement and deformation of bridge support elements).

#### 4.1.1. Semi-Automated Feature Extraction

**Figure 6:**
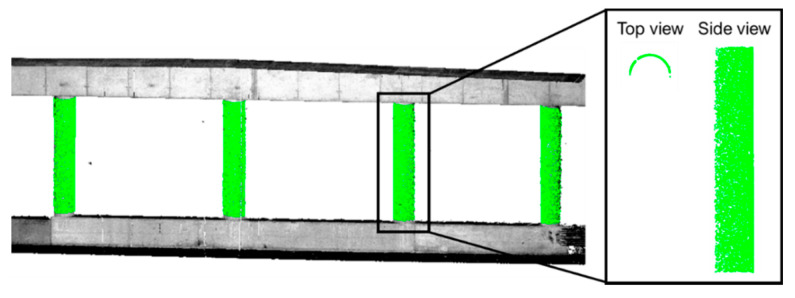
An example of cylindrical features (in green) that have been segmented from a LiDAR point cloud (colored by intensity).

The proposed feature extraction strategy is adapted from the multi-class simultaneous segmentation proposed by Habib and Lin [82]. First, a seed point is manually identified in the point cloud from the individual scans/tracks. If the point clouds are roughly aligned, the same seed point can be used to extract the feature from all scans/tracks. Otherwise, the user will need to select the seed point for each scan/track. Next, a seed region is established by identifying the neighboring points of the seed point. Dimensionality analysis [83] is then performed to classify the seed region as a linear/cylindrical, planar, or rough feature. Only planar, linear, and cylindrical seed regions are considered for feature extraction. The parameters of the best-fitting plane/line/cylinder, which will be discussed later in this section, are estimated via an iterative model fitting and outlier removal. More specifically, model parameters are estimated in an iterative manner while assigning lower weights to points that are farther from the fitted surface in the previous iteration. Next, neighboring points that belong to the current feature are augmented through region growing. Concretely, neighboring points whose normal distance are smaller than a multiplication factor times the root-mean-square error (RMSE) of the fitted model are added. The output of the feature extraction includes the segmented points (an example is shown in Figure 6) and parameters describing the respective feature model—both are required in the LSA registration and model parameterization.

**Figure 7:**
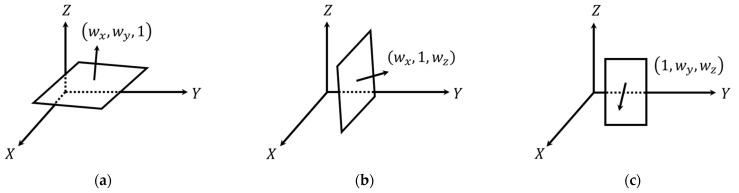
Different options for representing planar features showing the normal vectors to the planes (defined by the eigenvector corresponding to the smallest eigenvalue) that are mainly along the (**a**) *Z*-axis (i.e., the eigenvector component along the *Z*-axis is larger than those along the *X* and *Y* axes), (**b**) *Y*-axis (i.e., the eigenvector component along the *Y*-axis is larger than those along the *X* and *Z* axes), and (**c**) *X*-axis (i.e., the eigenvector component along the *X*-axis is larger than those along the *Y* and *Z* axes).

**Figure 8:**
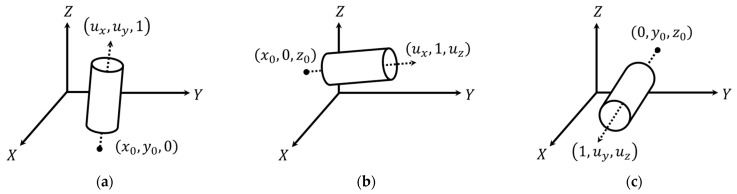
Different options for representing cylindrical features with directions (defined by the eigenvector corresponding to the largest eigenvalue) that are mainly along the (**a**) *Z*-axis (i.e., the eigenvector component along the *Z*-axis is larger than those along the *X* and *Y* axes), (**b**) *Y*-axis (i.e., the eigenvector component along the *Y*-axis is larger than those along the *X* and *Z* axes), and (**c**) *X*-axis (i.e., the eigenvector component along the *X*-axis is larger than those along the *Y* and *Z* axes).

In terms of the parametric model representation, a 3D plane is defined by the normal vector to the plane, ${\left[\begin{array}{ccc} w_{x} & w_{y} & w_{z} \end{array}\right]}^{T}$, and signed normal distance from the origin to the plane (d) as shown in Equation (1). To establish an independent set of parameters, one of the normal vector components is fixed to 1. The fixed component is chosen according to the orientation of the plane, which is defined based on the eigenvectors corresponding to the smallest eigenvalue, as illustrated in Figure 7. A cylindrical feature, on the other hand, is defined by a 3D line representing its axis and radius  (r). The cylinder axis is represented by a point, ${\left[\begin{array}{ccc} x_{0} & y_{0} & z_{0} \end{array}\right]}^{T}$, and a direction vector, ${\left[\begin{array}{ccc} u_{x} & u_{y} & u_{z} \end{array}\right]}^{T}$, as can be seen in Equation (2), where q represents the point location along the cylinder axis. Since a line has only four degrees of freedom, one of the coordinate components is set to zero, with the respective direction vector component set to 1. The fixed parameters for the cylinder axis are chosen according to its orientation, which is defined based on the eigenvectors corresponding to the largest eigenvalue, as depicted in Figure 8. Finally, a linear feature is represented the same way as the axis of a cylindrical feature (i.e., four parameters, with two representing a point along the line and two defining its direction).

(1) $$\frac{w_{x}x+w_{y}y+w_{z}z}{\sqrt[{}]{w_{x}^{2}+w_{y}^{2}+w_{z}^{2}}}+d=0$$

(2) $$\begin{array}{c} x=qu_{x}+x_{0}\\ y=qu_{y}+y_{0}\\ z=qu_{z}+z_{0} \end{array}$$

#### 4.1.2. Least-Squares Adjustment for Feature-Based Fine Registration

The conceptual basis of the proposed LSA model is that conjugate features would fit a single parametric model after registration. The objective function of the LSA model estimates the transformation parameters as well as feature parameters in the common reference frame. For simultaneous registration between $m_{s}$ point clouds, one of them is selected to define a common reference frame, (${\vec{pc}}_{c}={\left[\begin{array}{ccc} x_{c} & y_{c} & z_{c} \end{array}\right]}^{T}$), and the rest are considered as sources, (${\vec{pc}}_{si}={\left[\begin{array}{ccc} x_{si} & y_{si} & z_{si} \end{array}\right]}^{T},\;i=1,\;2,\;\ldots ,\;m_{s}-1$). The 3D similarity model in Equation (3) is used to represent the transformation from the $i^{th}$ source (si) to the common (c) reference frames, where ${\vec{t}}_{si}^{c},\;R_{si}^{c},s_{si}^{c}$ denote the translation vector, rotation matrix, and scale factor, respectively:

(3) $${\vec{pc}}_{c}={\vec{t}}_{si}^{c}+s_{si}^{c}R_{si}^{c}{\vec{pc}}_{si}$$

**Figure 9:**
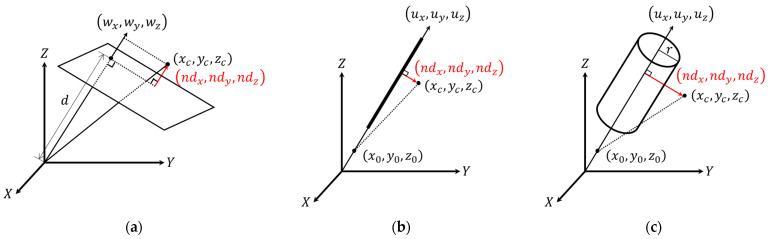
Schematic illustration of the normal distance vector components that are minimized using the (**a**) plane-fitting model, (**b**) 3D line-fitting model, and (**c**) cylinder-fitting model.

For planar features, the normal distance vector, ${\left[\begin{array}{ccc} nd_{x} & nd_{y} & nd_{z} \end{array}\right]}^{T}$, between a transformed point, ${\left[\begin{array}{ccc} x_{c} & y_{c} & z_{c} \end{array}\right]}^{T}$, and the post-registration parametric model, defined by ${\left[\begin{array}{ccc} w_{x} & w_{y} & w_{z} \end{array}\right]}^{T}$ and d, is presented in Equation (4)—refer to the illustration in Figure 9a. For a linear/cylindrical feature, the normal distance vector, ${\left[\begin{array}{ccc} nd_{x} & nd_{y} & nd_{z} \end{array}\right]}^{T}$, between a transformed point, ${\left[\begin{array}{ccc} x_{c} & y_{c} & z_{c} \end{array}\right]}^{T}$, and the post-registration parametric model representing the linear/axis of a cylindrical feature, defined by ${\left[\begin{array}{ccc} x_{0} & y_{0} & z_{0} \end{array}\right]}^{T}$ and ${\left[\begin{array}{ccc} u_{x} & u_{y} & u_{z} \end{array}\right]}^{T}$, is expressed in Equation (5). An illustration of the linear/cylindrical feature post-registration model fitting is shown in Figure 9b,c, respectively. For planar and linear features, the LSA aims at minimizing the squared sum of normal vector components in Equations (4) and (5), respectively, for all the points in the different scans/tracks that encompass such features. In these equations, ⊙ denotes the dot product of two vectors. For cylindrical features, on the other hand, the LSA aims at minimizing the squared sum of normal distances between the points that belong to such features and their surface, as given by Equation (6):

(4) $$\left[\begin{array}{c} nd_{x}\\ nd_{y}\\ nd_{z} \end{array}\right]=-\frac{d\left[\begin{array}{c} w_{x}\\ w_{y}\\ w_{z} \end{array}\right]}{\sqrt[{}]{w_{x}^{2}+w_{y}^{2}+w_{z}^{2}}}-\frac{\left(\left[\begin{array}{c} x_{c}\\ y_{c}\\ z_{c} \end{array}\right]⊙\left[\begin{array}{c} w_{x}\\ w_{y}\\ w_{z} \end{array}\right]\right)\left[\begin{array}{c} w_{x}\\ w_{y}\\ w_{z} \end{array}\right]}{w_{x}^{2}+w_{y}^{2}+w_{z}^{2}}=\left[\begin{array}{c} 0\\ 0\\ 0 \end{array}\right]$$

(5) $$\left[\begin{array}{c} nd_{x}\\ nd_{y}\\ nd_{z} \end{array}\right]=\left[\begin{array}{c} x_{c}-x_{0}\\ y_{c}-y_{0}\\ z_{c}-z_{0} \end{array}\right]-\frac{\left(\left[\begin{array}{c} x_{c}-x_{0}\\ y_{c}-y_{0}\\ z_{c}-z_{0} \end{array}\right]⊙\left[\begin{array}{c} u_{x}\\ u_{y}\\ u_{z} \end{array}\right]\right)\left[\begin{array}{c} u_{x}\\ u_{y}\\ u_{z} \end{array}\right]}{u_{x}^{2}+u_{y}^{2}+u_{z}^{2}}=\left[\begin{array}{c} 0\\ 0\\ 0 \end{array}\right]$$

(6) $$nd_{x}^{2}+nd_{y}^{2}+nd_{z}^{2}-r^{2}=0$$

**Figure 10:**
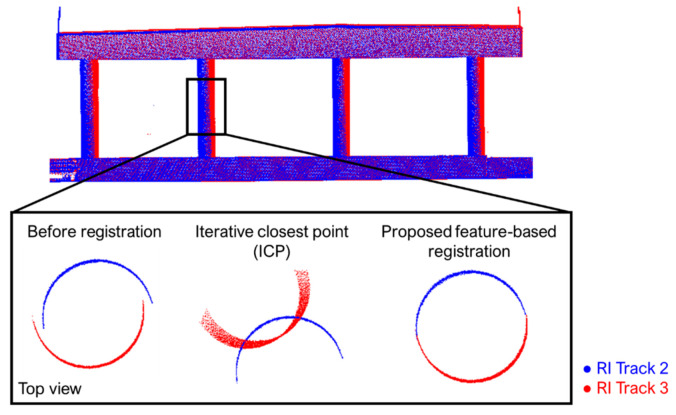
Sample registration results using the iterative closest point (ICP) and proposed approach, showing a cylindrical feature before and after registration.

The linearized mathematical model corresponding to Equations (4)–(6) can be written as in Equation (7), which is commonly known as the Gauss–Helmert model [84]. Here, y is the discrepancy vector arising from the linearization process; e is the vector of random noise contaminating the point cloud coordinates in the different scans/tracks, which follows a stochastic distribution with a zero mean and a variance–covariance matrix of $\sigma_{0}^{2}P^{-1}$, with $\sigma_{0}^{2}$ representing the a priori variance factor and the weight matrix, P, depending on the specifications of the data acquisition system; and x is the vector of unknown parameters (including transformation and feature parameters). The matrices A and B are composed of the partial derivatives of the models in Equations (4)–(6) with respect to the unknown parameters and point cloud coordinates, respectively. For a planar or linear feature, the respective B matrix is rank-deficient, which can be deduced by analyzing the effective contribution of Equations (4) and (5) towards the overall redundancy. Although a point on a planar feature provides three equations, it only has a net contribution of one towards the redundancy—corresponding to the minimization of normal distance to the plane. For linear features, the three equations have a net contribution of two towards the redundancy—corresponding to the minimization of normal distance to the linear feature. Lastly, a point on a cylindrical feature provides a net contribution of one towards the redundancy. In order to ensure that the resulting normal matrix is full-rank and that the transformation and feature parameters can be reliably estimated, it is critical to have features with different orientations over the area of interest. The solution vector of the LSA model can be written as per Equation (8), where ${\left(BP^{-1}B^{T}\right)}^{+}$ denotes the Moore–Penrose pseudoinverse that has to be used to compensate for the rank deficiency of the B matrix. The residuals, $\tilde{e}$, are given in Equation (9). The a posteriori variance factor, ${\hat{\sigma_{0}}}^{2}$, can be computed using Equation (10), where m is the number of unknown parameters. The variance–covariance matrix of estimated parameters is given in Equation (11). Interested readers can refer to Ravi and Habib [85] for more details regarding LSA with a rank-deficient weight matrix. Figure 10 presents a sample registration result using the ICP and proposed feature-based registration approach. The point clouds were acquired from two tracks in opposite driving directions, and thus capture different sides of the bridge piers. While the ICP incorrectly aligned the two sides of the piers, the proposed approach produced superior results by fitting conjugate features to a single cylinder model:

(7) $$y=Ax+Be,\;e\sim \;\left(0,\;\sigma_{0}^{2}P^{-1}\right)$$

(8) $$\hat{x}={\left(A^{T}{\left(BP^{-1}B^{T}\right)}^{+}A\right)}^{-1}\left(A^{T}{\left(BP^{-1}B^{T}\right)}^{+}y\right)$$

(9) $$\tilde{e}=y-A\hat{x}$$

(10) $${\hat{\sigma_{0}}}^{2}=\frac{{\tilde{e}}^{T}{\left(BP^{-1}B^{T}\right)}^{+}\tilde{e}}{rank\left(BP^{-1}B^{T}\right)-m}$$

(11) $$\Sigma ={\hat{\sigma_{0}}}^{2}\;{\left(A^{T}{\left(BP^{-1}B^{T}\right)}^{+}A\right)}^{-1}$$

### 4.2. Bridge Deck Thickness Evaluation

**Figure 11:**
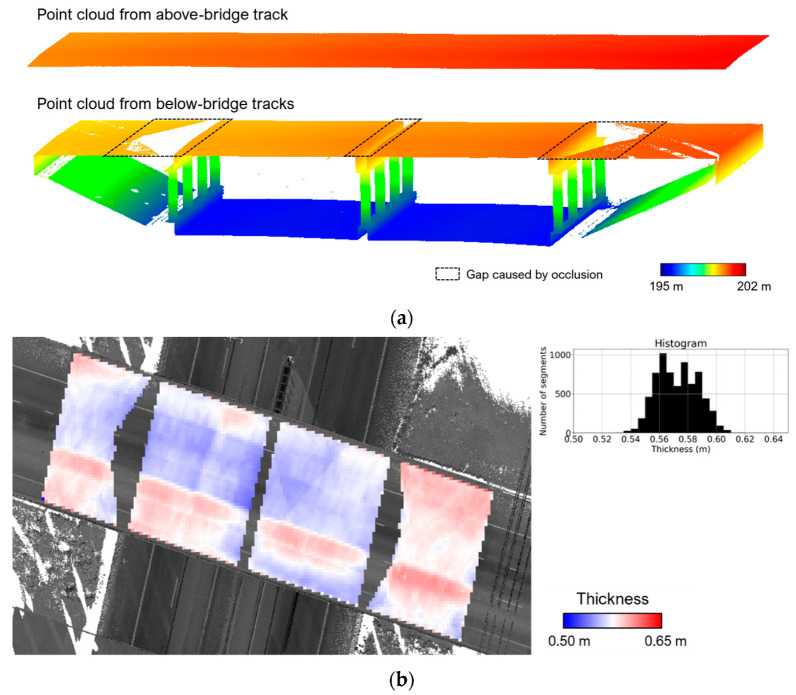
Example of (**a**) a point cloud capturing the top and bottom surfaces of the bridge deck and (**b**) a heat map representing the estimated bridge deck thickness.

The proposed bridge deck thickness evaluation utilizes surface-to-surface distance in order to mitigate the impact of inherent noise on the post-registration point cloud. First, point clouds capturing the top and bottom surfaces of the deck are extracted—one is selected as the target and another as the source. The target and source point clouds are then partitioned into segments with a pre-defined size (30 cm × 30 cm in this study). For a given segment, the dimensionality analysis is carried out to test its planarity, and only planar segments are included in subsequent evaluation. An iterative plane fitting is then performed to remove potential outlier points and estimate the parameters of the plane (refer to Section 4.1.1 for detailed information). A segment is rejected from the thickness evaluation if (i) the plane-fitting RMSE exceeds a user-defined threshold ($thres_{RMSE}$) or (ii) the remaining inlier points after iterative plane fitting fail to reach another user-defined threshold ($thres_{npt}$). The first threshold ($thres_{RMSE}$) can be selected according to the expected noise level in the post-registration point cloud. The second threshold ($thres_{npt}$) can be defined based on the percentage of remaining points after iterative plane fitting—e.g., if more than 50% of the original points are removed by the outlier removal procedure, the segment is rejected from the thickness evaluation. Next, the center of a target plane and its projection on the source plane are determined. The thickness of the deck at that location is evaluated by the normal distance between the center of the target plane and the source plane. Finally, the deck thickness estimates from all the surface segments are visualized as a heat map. Figure 11 illustrates an example, showing the point clouds from the above- and below-bridge tracks (Figure 11a) as well as the corresponding thickness map (Figure 11b). As evident in Figure 11a, the point cloud from the below-bridge tracks shows some gaps because of the occlusion caused by the bridge pier caps.

### 4.3. Comparative Quality Assessment

In this study, the derived bridge deck thickness using MLMS data is compared to that estimated from TLS point clouds. The comparison is carried out by evaluating the difference between the thickness estimates for corresponding surface segments in the MLMS and TLS data. Therefore, ensuring the alignment between MLMS and TLS data is a prerequisite. A coarse registration is conducted to transform the TLS point cloud into the MLMS mapping frame. Since the proposed segment-based thickness evaluation is conducted at 30 cm intervals and the coarse registration accuracy is better than ±10 cm, a fine registration procedure is not necessary. Once the point clouds are aligned, the correspondence is established by searching for the closest surface segment in the MLMS point cloud for each segment in the TLS point cloud. The difference between bridge deck thickness estimates for corresponding surface segments is evaluated and visualized as a heat map. Moreover, the mean, standard deviation, and RMSE of the differences are reported as quantitative measures of the comparative performance of the different sensing modalities (i.e., TLSs and MLMSs).

## 5. Experimental Results and Discussion

This section starts with reporting the registration results for the MLMS and TLS datasets. For the MLMS datasets, the alignment between point clouds from different tracks before and after registration is examined to illustrate the quality of the system calibration and the impact of GNSS outage during data acquisition. Next, the bridge deck thickness is evaluated, and a comparative analysis of the derived metrics is reported.

### 5.1. Point Cloud Registration and Alignment

**Figure 12:**
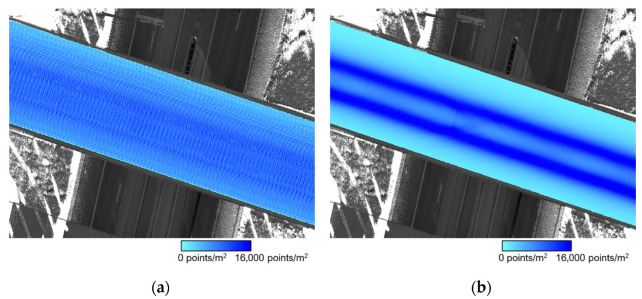
Point density over the bridge deck from one track (track T1) for: (**a**) PWMMS-HA and (**b**) PWMMS-UHA.

As described in Section 3.2, one TLS and two MLMS datasets were collected in this study. The TLS dataset had six scans, with each having its local coordinate system. First, a coarse registration using manually identified point pairs in TLS point clouds was performed. For such registration, one of the FARO scans, Scan S1 (see Figure 3), was selected to define the common reference frame. In contrast to the TLS data, point clouds acquired by the MLMSs were directly georeferenced to a global mapping frame through the onboard GNSS/INS unit—the UTM coordinate system with WGS84 as the datum was chosen as the reference frame in this study. The point density over the bridge deck from one track is depicted in Figure 12, where the median is 7700 and 6800 points per square meter for PWMMS-HA and PWMMS-UHA, respectively. PWMMS-HA had a higher and more uniform point density across the bridge deck as compared to PWMMS-UHA. 

**Figure 13:**
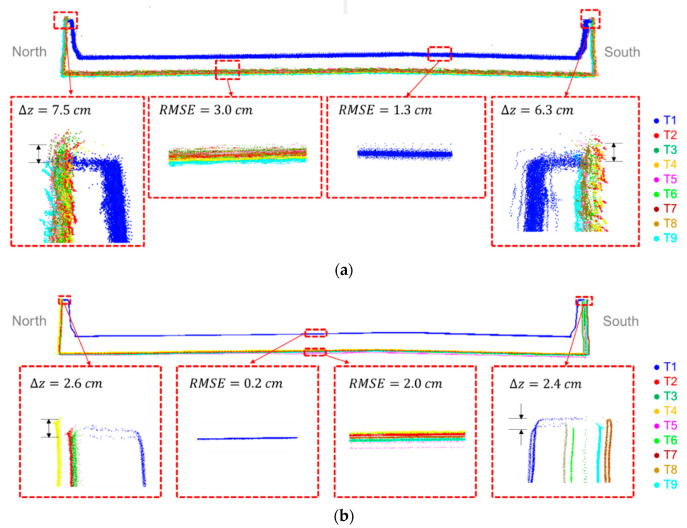
Sample cross-sectional profile of the bridge deck showing the point cloud alignment before registration for the (**a**) PWMMS-HA and (**b**) PWMMS-UHA datasets. Both datasets have nine tracks (T1 is above the bridge and T2–T9 are below the bridge).

Prior to fine registration, the alignment of MLMS point clouds from different tracks was inspected. Figure 13 depicts a cross-section of the bridge deck before registration. The top surface of the bridge deck and inside surface of the barrier rails were captured by the above-bridge track (T1), while the bottom surface of the bridge deck and the outside surface of the barrier rails were scanned by the below-bridge tracks (T2–T9). To assess the agreement between point clouds acquired by the four LiDAR units onboard the PWMMS-HA, we performed plane fitting over a 1 m × 1 m segment on the top surface of the bridge deck (see Figure 13a). The plane-fitting RMSE was 1.3 cm, indicating that the point clouds from different LiDAR units in Track T1 were in good agreement, thus verifying the quality of the system calibration parameters (i.e., inaccurate system calibration would result in discrepancies among captured point clouds by different sensors in the same track). A segment on the bottom surface of the bridge deck, in contrast, yielded a plane-fitting RMSE of 3.0 cm. Since this area was captured by eight tracks (T2–T9), one can deduce that the alignment among the point clouds from those tracks was not as good as the alignment between point clouds captured by the PWMMS-HA LiDAR sensors within a given track. The lower trajectory quality due to intermittent access to a GNSS signal below the bridge was the cause of such systematic discrepancy among the tracks, as can be seen in Figure 13a. A similar analysis was conducted for the PWMMS-UHA point clouds, as can be seen in Figure 13b. The zoomed-in area on the top surface of the bridge deck reveals that for a given track, the precision of point clouds from the two LiDAR units onboard the PWMMS-UHA was in the range of few millimeters. However, due to the intermittent access to a GNSS signal during below-bridge data acquisition, a degradation in the RMSE to the 2.0 cm range can be seen. The barrier rails along the two sides of the bridge were used to evaluate the alignment between the above-bridge and below-bridge tracks. The misalignment was interactively quantified by evaluating the distance between two manually selected points on the top of the barrier rail from the above-bridge and below-bridge point clouds. As shown in Figure 13a,b, a misalignment of about 7 cm and 2 cm along the vertical direction was present between the above-bridge and below-bridge point clouds for the PWMMS-HA and PWMMS-UHA, respectively. Such misalignment would lead to, no doubt, an unreliable estimation of bridge deck thickness.

**Figure 14:**
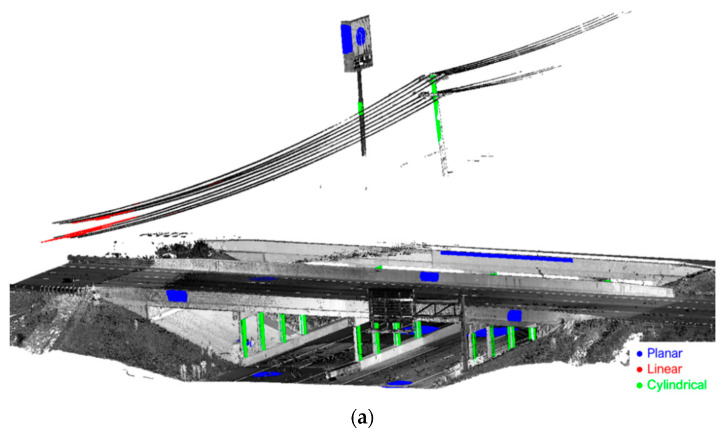

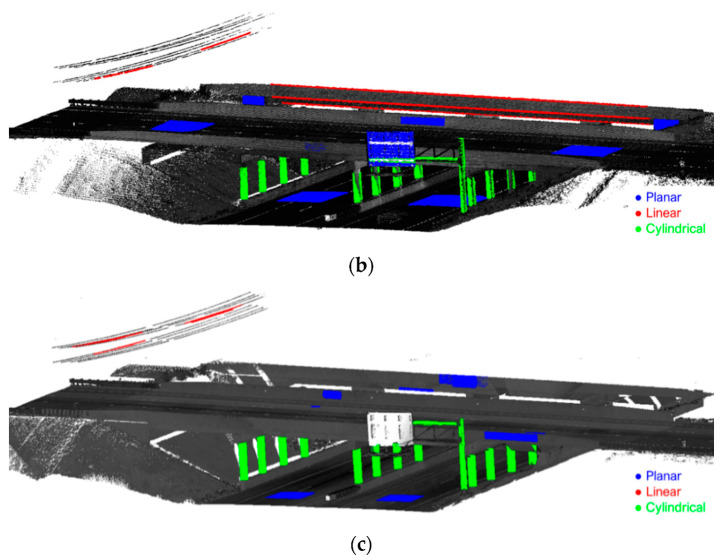
Extracted planar/linear/cylindrical features (in blue/red/green, respectively) for the registration of (**a**) TLS, (**b**) PWMMS-HA, and (**c**) PWMMS-UHA point clouds.

The proposed feature-based fine registration was then performed for the TLS and MLMS datasets to refine the alignment between scans/tracks. First, planar/linear/cylindrical features were semi-automatically extracted from the point clouds, and the results are shown in Figure 14. In order to reliably solve for the transformation parameters, features with different orientations were extracted from the point clouds. These features should be well-distributed over the area of interest, and the number of points contributing to the estimation of different transformation parameters was of similar magnitude to prevent over-weighting in the LSA model. A total of 46, 30, and 29 features were extracted from the TLS, PWMMS-HA, and PWMMS-UHA point clouds, respectively. For the TLS dataset, the overlap between the above-bridge and below-bridge scans was adequate, as the Trimble scanner was placed on the I-74 embankments next to the barrier rails, and thus was able to capture objects on the US-231 without much occlusion. For the MLMS datasets, on the other hand, the overlap between the above- and below-bridge tracks was limited due to occlusions caused by the barrier rails. Point clouds acquired by the above-bridge track barely captured the road surface, signboards, and other objects on the US-231. Common features among the above-bridge and below-bridge tracks include signboards, light poles, and the north side of the barrier rail on the westbound I-74, as can be seen in Figure 14b,c.

**Figure 15:**
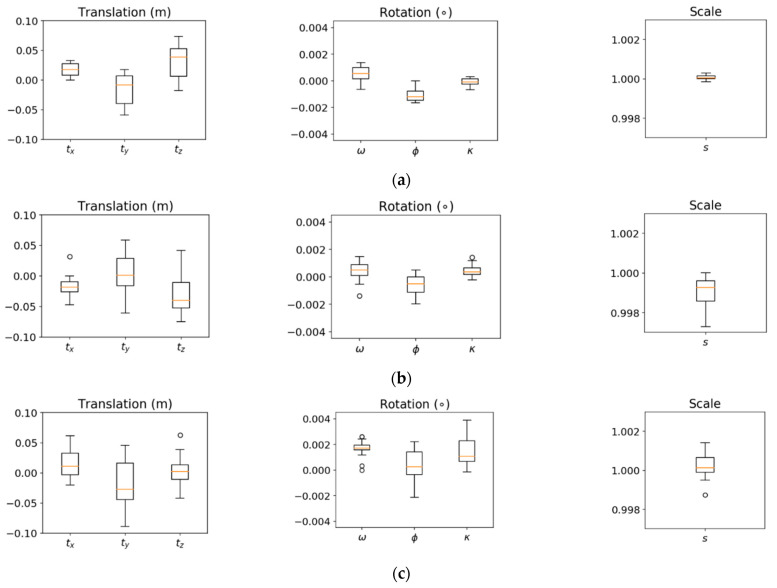
Box and whisker plots of the fine-registration transformation parameters for the (**a**) TLS, (**b**) PWMMS-HA, and (**c**) PWMMS-UHA datasets.

Once the features were extracted, the proposed LSA strategy was carried out to estimate the transformation and post-registration feature parameters. For the MLMS datasets, the LSA estimated the transformation parameters among point clouds acquired by each sensor from individual tracks (i.e., the registration was simultaneously conducted for a total of thirty-six and eighteen point clouds for PWMMS-HA and PWMMS-UHA, respectively). Track T1 from HDL-RR and Track T1 from RI were selected as target tracks for PWMMS-HA and PWMMS-UHA, respectively. For TLSs, Scan S1 was used as the target scan when registering the six point clouds captured by the FARO and Trimble scanners. Figure 15 shows box and whisker plots of the transformation parameters for the TLS and MLMS datasets, where one can observe that the magnitude and variance of the parameters for each system were at the same level. The square root of a posteriori variance factor after registration (which represents the noise level in the data as well as the quality of post-registration alignment) is 0.60 cm, 1.47 cm, and 0.74 cm for TLSs, PWMMS-HA, and PWMMS-UHA, respectively. The weighted average of the RMSE of normal distances between the LiDAR points and the best-fitted plane/line/cylinder before and after registration for each dataset is listed in Table 2. The reduction in the post-registration RMSE reflects an improvement in the alignment of the features after registration. As expected, the post-registration RMSE values for the different features are in agreement with the square root of a posteriori variance.

**Figure 16:**
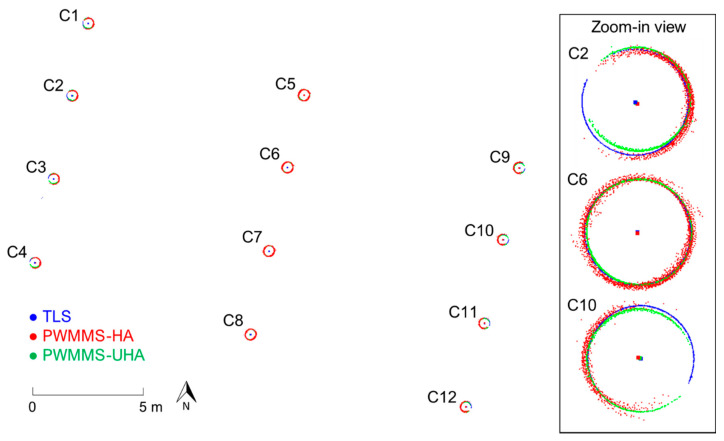
The twelve cylindrical columns and their axes derived from TLS (in blue), PWMMS-HA (in red), and PWMMS-UHA (in green) data.

One of the advantages of the proposed alignment strategy is the production of a parametric model representation of the registration primitives, which could be used for bridge monitoring over time (e.g., the cylindrical columns supporting the bridge). To show the comparative performance of TLS and MLMSs in terms of the similarity of derived post-registration parametric models for cylindrical features, Figure 16 depicts a top view of the twelve columns supporting the I-74 bridge together with their axes. As evident from the figure, the cylindrical columns from different sensing modalities are well-aligned. Table 3 reports the estimated radii of the twelve columns where the estimates from different systems are in agreement within the 1 cm range. Moreover, the relative planimetric discrepancies between the TLS and MLMS datasets were evaluated using the derived horizontal locations of the cylindrical columns. The results show that the relative horizontal locations are compatible within a 1 cm range. Once again, these values are in agreement with the post-registration estimates of the square root of a posteriori variance factors reported earlier.

**Figure 17:**
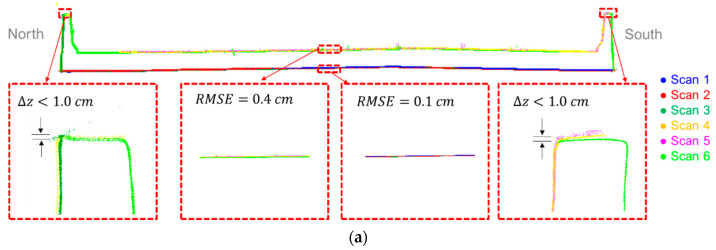

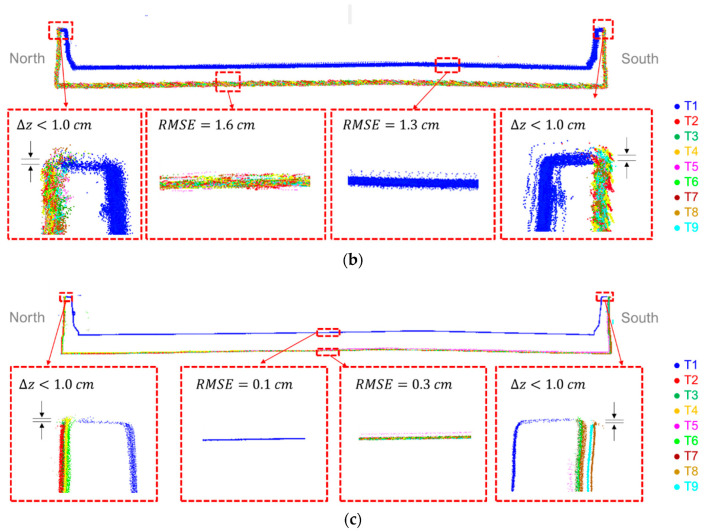
Sample cross-sectional profile showing the post-registration point cloud alignment for the (**a**) TLS, (**b**) PWMMS-HA, and (**c**) PWMMS-UHA datasets.

As another verification of the impact of the fine registration of the MLMS point clouds, a cross-section of the bridge deck (at the same location shown in Figure 13) that was extracted from the point is depicted in Figure 17, where a significant improvement can be observed. Zoomed-in areas at the barrier rails show that the above-bridge and below-bridge tracks/scans are in agreement within a 1 cm range for all systems. Moreover, zoomed-in areas at the top and bottom surfaces of the bridge deck verify that the point clouds from different tracks/scans are in good agreement along the vertical direction. According to the plane-fitting RMSE shown in Figure 17, the precision of the point cloud from PWMMS-HA is in the ±1.5 cm range, which is better than the expected value of ±4 cm. Moreover, both PWMMS-UHA and TLS point clouds achieve millimeter-level precision (i.e., in the ±0.3 cm and ±0.4 cm range, respectively). In summary, the results show that the proposed feature-based fine registration can effectively minimize the impact of trajectory and system calibration errors, and thus improve the point cloud quality.

### 5.2. Bridge Deck Thickness Estimation and Comparative Analysis

**Figure 18:**
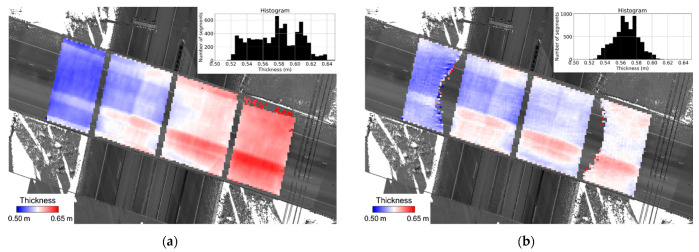

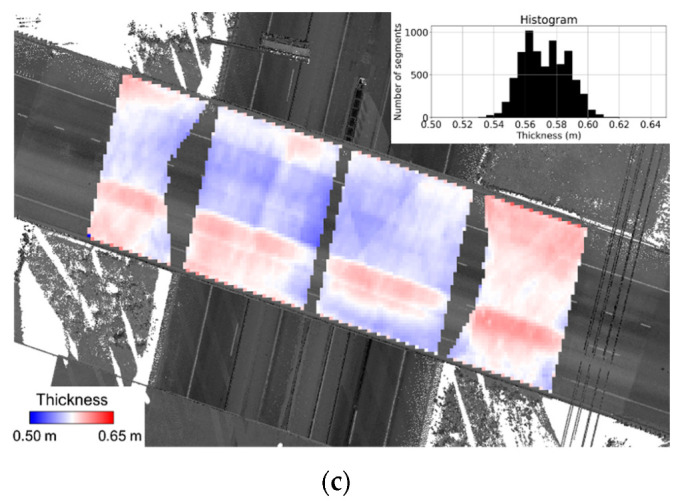
Bridge deck thickness estimates shown as a heat map using the (**a**) TLS, (**b**) PWMMS-HA, and (**c**) PWMMS-UHA datasets.

Having examined the point cloud alignment, bridge deck thickness was then evaluated using the proposed surface segment-based approach. The segment size was set to 30 cm × 30 cm. The thresholds $thres_{RMSE}$ and $thres_{npt}$ were defined as three times the square root of a posteriori variance factor after registration and 50% of it, respectively. The thickness estimates are visualized as a heat map and shown in Figure 18. The spatial patterns from the three datasets are similar as they all indicate a smaller thickness value over the right two lanes towards the west side of the bridge.

**Figure 19:**
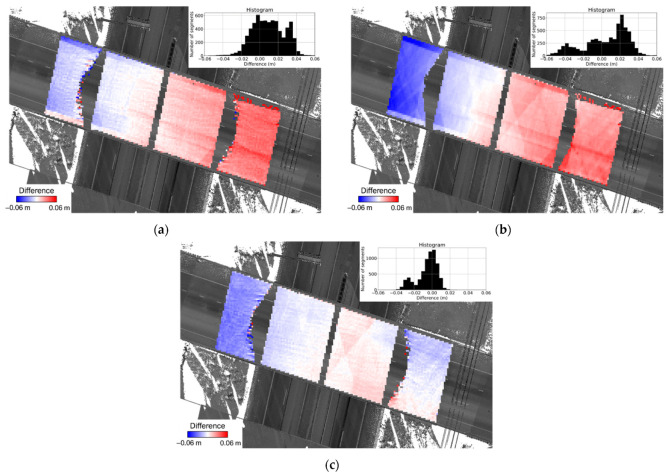
Heat map visualization of the difference in bridge deck thickness estimates between (**a**) TLS and PWMMS-HA, (**b**) TLS and PWMMS-UHA, and (**c**) PWMMS-HA and PWMMS-UHA.

To quantify the similarity of thickness estimates using different modalities, a coarse registration between the TLS and MLMS datasets was performed to align the former to the reference frame of the latter. The registration accuracy was better than ±10 cm, which is good enough for identifying corresponding surface segments. The difference between the thickness estimates at each segment were evaluated and visualized as a heat map, as can be seen in Figure 19. The mean, standard deviation, and RMSE of the thickness differences are reported in Table 4. One should note that although the PWMMS-HA point cloud is less accurate, it has a higher point density, which in turn provides larger redundancy for plane fitting in bridge deck thickness evaluation. Therefore, the thickness evaluation accuracy for PWMMS-HA is similar to that for PWMMS-UHA. The results show that the bridge deck thickness estimates from different systems are in agreement within the 1–3 cm range. A closer investigation of the results in Figure 19 and Table 4 reveals that there is a higher level of compatibility in the thickness estimates from the PWMMS-HA and PWMMS-UHA systems. When compared to the TLS-based thickness estimates, one can observe a trend in the difference (i.e., underestimation/overestimation of thickness at the west side/east side of the bridge, respectively). This trend is the result of the Trimble scan locations leading to less-than-optimal overlap between the east and west above-bridge scans. Therefore, it is believed that the variation in the thickness estimate is in the 1 cm range. In summary, the PWMMS-HA, although with a centimeter-level accuracy LiDAR unit, has a similar performance as the PWMMS-UHA. The derived thickness from the MLMS units is comparable to that derived using TLS, with the latter being more sensitive to the scan locations. The results also reveal that the proposed segment-based thickness evaluation can handle inherent noise in the point cloud and provide reliable thickness estimates.

## 6. Conclusions and Recommendations for Future Work

This paper presented an evaluation of the performance of mapping-grade and surveying-grade mobile LiDAR systems for bridge monitoring. The performance of these systems was assessed against static laser scanners. To take full advantage of MLMS-based point clouds, a semi-automated feature-based fine registration was proposed to mitigate the negative impact of georeferencing and system calibration errors. The proposed procedure can simultaneously estimate the necessary transformation parameters for the alignment of all the derived point clouds by various sensors onboard the MLMS from different tracks. In addition, the post-alignment parametric model of the registration primitives (planar, linear, and cylindrical features) is also estimated. Bridge deck thickness was evaluated using surface segments while minimizing the impact of inherent noise in the point clouds. Field surveys were carried out over a representative bridge that had grinding conducted on it to achieve desired pavement smoothness and ride quality. The results show that the proposed feature-based fine registration effectively mitigated the impact of intermittent accessibility to a GNSS signal below the bridge. The post-registration alignment quality for the point clouds captured by the mapping-grade MLMS, surveying-grade MLMS, and TLS units is ±1.5 cm, ±0.7 cm, and ±0.6 cm, respectively. Although point clouds from the mapping-grade system had a higher noise level, the evaluated bridge deck thickness was compatible to the one derived from the surveying-grade system in the range of 1 cm. The thickness estimates from both MLMS units were compatible with that derived from the TLSs. The MLMS data acquisition was conducted in five minutes, while TLSs took more than three hours. The proposed fine-registration strategy also delivered a parametric model of bridge elements, which can be used for monitoring the bridge elements over time. MLMS-based parametric models are in agreement with those from the TLSs in the 1 cm range.

Future research will focus on improving the automation level of feature extraction, as well as deriving other quantitative measures for the identification of structural issues. Moreover, larger infrastructure will be inspected to evaluate the impact of extended outage in GNSS signal reception on derived MLMS point clouds. Finally, we will be focusing on automated segmentation and parametric model representation of different structural elements of the infrastructure to aid the periodic monitoring process.

## Figures and Tables

**Table 1:** Specifications of the acquired point clouds by PWMMS-HA, PWMMS-UHA, and Terrestrial laser scanners (TLSs) above and below the bridge in question.

	Sensor	Number of Tracks/Scans	Number of Points per Track/Scan	Data Acquisition Time
PWMMS-HA	HDL-RR	9	~7 million	5 min
	HDL-RL	9	~7 million	
	HDL-FL	9	~7 million	
	VLP-FR	9	~2 million	
PWMMS-UHA	RI	9	~15 million	5 min
	ZF	9	~15 million	
TLS	FARO	3	~167 million	3 h
	Trimble	3	~199 million	

**Table 2:** Weighted average of the RMSE of plane/line/cylinder fittings before and after registration for TLS, PWMMS-HA, and PWMMS-UHA.

		TLS	PWMMS-HA	PWMMS-UHA
Number of features	Planar	19	11	10
	Linear	6	5	4
	Cylindrical	21	14	15
	Total	46	30	29
Weighted average of RMSE (m)	Before	0.014	0.026	0.029
	After	0.004	0.014	0.007

**Table 3:** Estimated post-registration radii of the cylindrical columns supporting the I-74 bridge from the TLS, PWMMS-HA, and PWMMS-UHA point clouds.

	Number of Points			Radius (m)			Difference (m)		
ID	TLS	HA	UHA	TLS	HA	UHA	TLS vs. HA	TLS vs. UHA	HA vs. UHA
C1	24,752	155,483	86,355	0.314	0.314	0.308	0.000	−0.006	−0.006
C2	25,764	150,622	80,216	0.305	0.305	0.297	0.000	−0.008	−0.008
C3	25,840	149,090	78,308	0.306	0.304	0.294	−0.002	−0.012	−0.010
C4	25,366	149,641	78,516	0.305	0.303	0.291	−0.002	−0.014	−0.012
C5	30,775	242,075	158,403	0.316	0.317	0.313	0.001	−0.003	−0.003
C6	20,143	234,633	156,096	0.305	0.306	0.302	0.001	−0.004	−0.004
C7	20,267	233,235	152,566	0.305	0.306	0.301	0.001	−0.004	−0.005
C8	20,123	231,872	149,291	0.305	0.306	0.301	0.001	−0.004	−0.005
C9	25,985	140,673	113,636	0.315	0.310	0.297	−0.005	−0.018	−0.012
C10	26,942	136,115	108,052	0.305	0.302	0.291	−0.003	−0.014	−0.011
C11	27,124	137,503	103,772	0.305	0.303	0.295	−0.002	−0.010	−0.008
C12	25,314	136,477	102,548	0.305	0.303	0.298	−0.002	−0.007	−0.006
						Mean	−0.001	−0.009	−0.008
						Std. Dev.	0.002	0.005	0.003
						RMSE	0.002	0.010	0.008

**Table 4:** Statistics of the difference between bridge deck thickness estimates from different systems.

	TLS vs. PWMMS-HA	TLS vs. PWMMS-UHA	PWMMS-HA vs. PWMMS-UHA
Mean (m)	0.010	0.005	−0.006
Std. Dev. (m)	0.018	0.025	0.012
RMSE (m)	0.021	0.025	0.013

## Data Availability

Data sharing is not applicable to this paper.
